# CircAMOTL1 promotes adipose lipolysis and browning in cancer cachexia through miR-211-5p-mediated TET2 activation

**DOI:** 10.1016/j.jbc.2025.111052

**Published:** 2025-12-12

**Authors:** Zuoyou Ding, Zhige Zhang, Jun Han, Ruizhao Dong, Ziang Yang, Guohao Wu, Qiulin Zhuang

**Affiliations:** Department of General Surgery, Zhongshan Hospital of Fudan University, Shanghai, People's Republic of China

**Keywords:** cancer cachexia, adipose tissue, circAMOTL1, TET2, lipolysis

## Abstract

Cancer cachexia is characterized by profound adipose tissue loss and metabolic remodeling, yet the regulatory mechanisms driving adipocyte dysfunction remain incompletely understood. Through whole-transcriptome sequencing of human subcutaneous adipose tissue, we identified circAMOTL1 as one of the most significantly upregulated circRNAs in patients with cachexia. circAMOTL1 expression positively correlated with weightloss severity and demonstrated strong diagnostic performance for cachexia. Functional studies revealed that circAMOTL1 promotes lipolysis and white adipose browning in adipocytes as evidenced by increased expression of ATGL, HSL, UCP1, and PGC-1α, elevated free fatty acid release, and enhanced mitochondrial content. Silencing circAMOTL1 produced the opposite phenotype. Mechanistically, circAMOTL1 localized predominantly in the cytoplasm and acted as a molecular sponge for miR-211-5p, thereby relieving miR-211-5p-mediated repression of TET2. Luciferase reporter, RIP, and FISH assays confirmed the circAMOTL1/miR-211-5p/TET2 regulatory interaction. Gain- and loss-of-function experiments demonstrated that TET2 is essential for circAMOTL1-induced lipolysis and thermogenic remodeling. *In vivo*, adipose-targeted recombinant AAV overexpression of circAMOTL1 in a C26 tumor-bearing mouse model induced adipose wasting and upregulated lipolytic and browning markers, phenocopying cachexia-associated adipose remodeling. These findings identify circAMOTL1 as a critical regulator of adipocyte metabolism in cancer cachexia. By modulating the circAMOTL1/miR-211-5p/TET2 axis, it drives lipolysis and thermogenic reprogramming of white adipose tissue. circAMOTL1 therefore represents a promising biomarker and a potential therapeutic target for preventing or attenuating cachexia-associated adipose tissue loss.

Cachexia represents a complex syndrome distinguished by pronounced involuntary weight reduction, predominantly resulting from the depletion of skeletal muscle and adipose tissue, in conjunction with metabolic abnormalities and systemic inflammation (1). It profoundly impacts quality of life and accounts for approximately 20% of cancer-related mortality. In recent years, researchers have shown growing interest in understanding how cachexia leads to the loss of adipose tissue. It has been demonstrated that the loss of adipose tissue generally transpires prior to the manifestation of other classical symptoms associated with cachexia (2). The reduction of adipose tissue in cachectic individuals encompasses not only heightened lipolysis but also considerable metabolic reprogramming, which includes the browning of white adipose tissue (WAT). This phenomenon transforms energy-storing white adipocytes into energy-expending brown-like adipocytes, characterized by the upregulation of thermogenic genes such as uncoupling protein 1 (UCP1) (3). Importantly, the latest investigations have clarified the pathological functions of molecules such as glucose-regulated protein 75 (GRP75) and macrophage migration inhibitory factor (MIF), revealing their roles in the adipose dysfunction observed in cachectic patients through various distinct mechanisms (4). GRP75 facilitates adipocyte browning *via* mitochondrial pathways, whereas MIF hinders adipogenesis by directing adipose progenitor cells toward pro-inflammatory and pro-fibrotic phenotypes, thereby underscoring novel therapeutic targets in the context of cancer-associated cachexia (5).

Circular RNAs (circRNAs) and microRNAs (miRNAs) represent classes of non-coding RNAs involved in numerous physiological and pathological processes by modulating gene expression at the transcriptional or post-transcriptional levels (6). CircRNAs, characterized by covalently closed-loop structures, function primarily as competitive endogenous RNAs (ceRNAs), or "miRNA sponges," thereby influencing the activity and availability of specific miRNAs (7). MiRNAs are short, endogenous non-coding RNAs that regulate gene expression by binding complementary sequences in the 3′ UTR of target mRNAs, leading to the degradation or translational inhibition. Emerging evidence has revealed significant roles for specific circRNAs and miRNAs in the pathogenesis of cachexia, particularly in adipose tissue and skeletal muscle wasting. For instance, miRNAs such as miR-27b-3p, miR-375, and miR-424-5p have been implicated in muscle and adipose tissue degradation by targeting genes associated with muscle atrophy and lipolysis pathways (8). It has been identified that circANAPC7 acts as a tumor suppressor that inhibits tumor growth and muscle wasting *via* the CREB-miR-373-PHLPP2 axis, leading to dephosphorylation of AKT and downregulation of cyclin D1 and TGF-β, thereby suppressing pancreatic cancer progression and cachexia (9). Presently, the role of non-coding RNAs in adipose tissue loss among cachexia patients remains largely unexplored. Understanding these non-coding RNAs' regulatory roles provides novel insights into cachexia's molecular basis and highlights their potential as biomarkers and therapeutic targets.

AMOTL1, a motin-family scaffold protein, contributes to epithelial polarity, migration, and junctional organization in diverse tissues and has been implicated in tumor progression and endothelial dynamics. Its circular isoform, circAMOTL1, has emerged as a versatile regulatory molecule with roles far beyond those of its linear host gene. In cardiovascular biology, circAMOTL1 is highly enriched in neonatal hearts and enhances cardiomyocyte survival by directly binding PDK1 and AKT, thereby promoting AKT phosphorylation, nuclear translocation, and protection against doxorubicin-induced injury (10). In cancer, circAMOTL1 exerts potent oncogenic functions: It is upregulated in cervical tumors, drives proliferation and migration, and modulates the AMOTL1-miRNA axis by sponging miR-485-5p to derepress AMOTL1 expression, ultimately accelerating tumor growth *in vitro* and *in vivo* (11). Collectively, these findings indicate that circAMOTL1 acts as a context-dependent effector capable of regulating kinase signaling, apoptosis, and cell motility, underscoring its broad relevance in tissue remodeling, tumor biology, and stress responses.

Ten-Eleven Translocation-2 (TET2) is an essential epigenetic regulator implicated in DNA demethylation processes. Emerging research has highlighted the significant role of TET2 mutations and their associations with metabolic diseases such as insulin resistance and obesity (12). Studies utilizing mouse models have revealed that TET2 loss-of-function mutations in hematopoietic cells exacerbate insulin resistance induced by aging and obesity. Additionally, endothelial TET2 has been identified as a crucial regulator of adipose tissue metabolism and vascularization in obesity. Endothelial-specific deficiency of TET2 results in impaired function of adipose tissue browning and exacerbation of diet-induced obesity (13). It is demonstrated that adipocyte TET2-regulated leptin gene expression through DNA demethylation, establishing a negative feedback loop with leptin. This mechanism contributes to the regulation of body weight and offers potential therapeutic insights for obesity management (14). These findings underscore TET2’s pivotal role in adipocyte biology and suggest that modulation of TET2 activity may represent a promising therapeutic approach for metabolic diseases. However, the involvement of TET2 in cachexia remains unexplored, underscoring its significance as a valuable subject for further investigation.

In the present study, through RNA sequencing, we have identified a circRNA derived from AMOTL1, termed circAMOTL1, significantly upregulated in the adipose tissues of cachectic patients compared to non-cachectic controls. We propose that the increased levels of circAMOTL1 may upregulate TET2 expression, thus promoting lipolysis and adipocyte browning by sponging miR-211-5p. Our findings suggest that circAMOTL1 acts as a promoter in cachexia progression and may offer a novel target for both the diagnosis and the treatment of cachexia.

## Results

### CircAMOTL1 is upregulated in adipose tissues of cachectic patients

**Figure 1:**
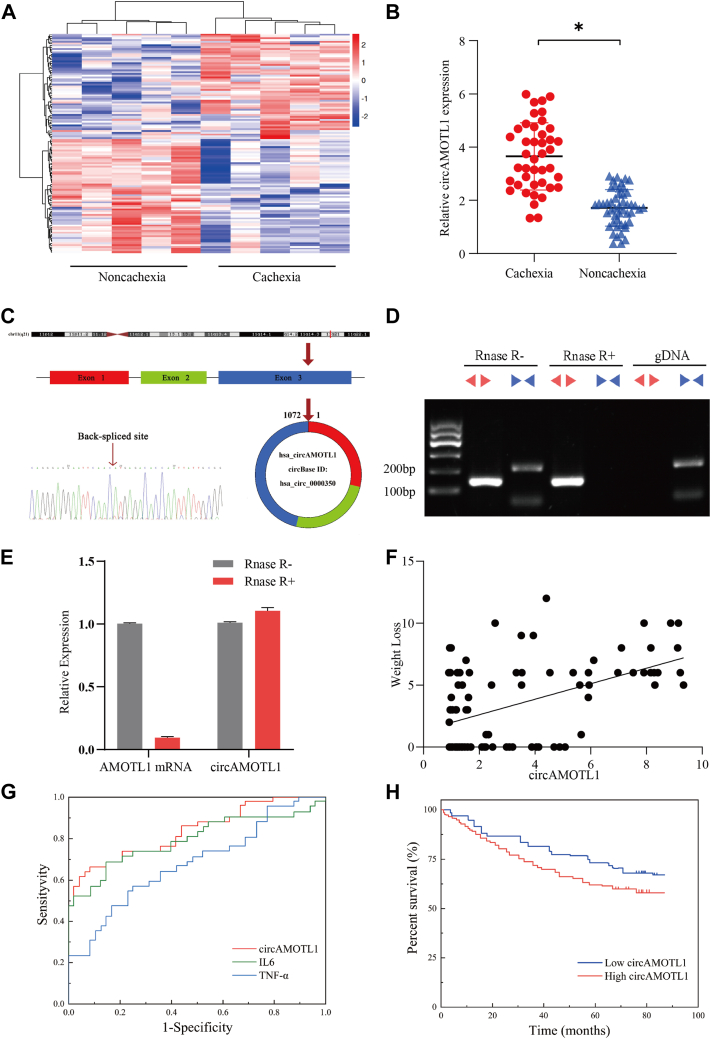
**CircAMOTL1 is upregulated in patients with cachexia and is correlated with cachexia-related clinical characteristics.***A*, heatmap of the differentially expressed circRNAs between patients with or without cachexia. *B*, relative expression of hsa_circ_0000350 verified in adipose tissue samples by qPCR. *C*, genomic loci of the circAMOTL1 gene. The back splicing junction was validated by Sanger sequencing. *D*, RT-PCR analysis of circAMOTL1 and AMOTL1 mRNA expression after RNase R treatment. Divergent primers detected circAMOTL1 from cDNA, but not from gDNA. *E*, qRT-PCR was used to determine the abundance of circAMOTL1 and AMOTL1 mRNA after treatment with RNase R in adipose tissue. *F*, correlation between circAMOTL1 expression and weight loss in 97 patients. *G*, the ROC curve concludes IL6, TNFα, and circAMOTL1 in distinguishing patients with cachexia and non-cachexia patients. *H*, the overall survival of 97 patients with high *versus* low circAMOTL1 levels is shown as K-M curve.

To identify adipose-associated circRNAs implicated in cachexia, we conducted whole-transcriptome RNA sequencing of subcutaneous WAT obtained from patients with and without cachexia. Comparative analysis revealed a distinct set of differentially expressed circRNAs between two groups. Among 398 differentially expressed circRNAs, 218 circRNAs were upregulated, whereas 180 circRNAs were downregulated in the cachexia group (|log_2_FC|>1, *p* < 0.05) (Fig. 1*A*). It was revealed that hsa_circ_0000350 was upregulated in the cachectic cohort. qPCR analysis of available adipose tissue specimens corroborated this finding, demonstrating that significantly higher expression of circAMOTL1 in the subcutaneous adipose tissue of patients with cachexia compared to non-cachectic controls (*p* < 0.05, Fig. 1*B*). According to the human reference genome (GRCh37/hg19), we identified that hsa_circ_0000350 is derived from exons of the AMOTL1 gene, which is located on chromosome 11q14.3. Sanger sequencing of the amplified PCR products confirmed the back-splicing sites of hsa_circ_0000350 (Fig. 1*C*). Then, we searched circBank (ID: hsa_AMOTL1_0000900) and circAtlas (ID: hsa-AMOTL1_0001) and found that mmu_circ_0000160 is the homologous RNA of hsa_circ_0000350. Hence, it convinced us that both circRNAs are homologous, and we used circAMOTL1 as their general term in the following articles. To assess the resistance of circAMOTL1 to exonuclease-mediated degradation, total RNA was subjected to RNase R treatment, with the abundance of linear isoforms serving as a control for digestion efficiency. To further validate the circular structure of circAMOTL1, we employed both convergent and divergent primers to amplify transcripts from complementary DNA (cDNA) and genomic DNA (gDNA) isolated from adipocytes. Divergent primers successfully amplified circAMOTL1 from cDNA but not from gDNA, thereby excluding the possibility that the back-splice junction arose from trans-splicing events or genomic rearrangements (Fig. 1*D*). Moreover, hsa_circ_0000350 exhibited pronounced resistance to RNase R digestion relative to linear AMOTL1 mRNA, corroborating its circular structure in adipocytes (Fig. 1*E*). We next investigated the clinical significance of circAMOTL1 expression in subcutaneous adipose tissues from patients with gastrointestinal tumor. Clinical characteristics of 97 patients were collected, and their serum determinations were tested before surgery (Table 1). To explore the clinical relevance of circAMOTL1, we performed a correlation analysis between its expression levels and weight loss. Notably, circAMOTL1 expression exhibited a positive correlation with the degree of weight loss among patients (r = 0.504, *p* = 0.003), suggesting a potential link between circAMOTL1 upregulation and cachexia severity (Fig. 1*F*). Receiver–operating characteristic (ROC) curve analysis was performed to evaluate the diagnostic potential of circAMOTL1 for cachexia. The analysis revealed that circAMOTL1 achieved an area under the curve (AUC) of 0.826 (95% CI: 0.746–0.915; *p* < 0.001), outperforming IL-6 (AUC = 0.812, 95% CI: 0.708–0.894; *p* < 0.001) and TNF-α (AUC = 0.691, 95% CI: 0.578–0.796; *p* < 0.001) (Fig. 1*G*). A circAMOTL1 expression threshold of 3.464 was identified as the optimal cut-off value. These findings suggest that circAMOTL1 holds promise as a diagnostic biomarker for distinguishing cachectic patients from non-cachectic controls. Furthermore, Kaplan-Meier survival analysis demonstrated that elevated circAMOTL1 expression in cachectic patients was significantly associated with reduced overall survival (Fig. 1*H*).

**Table 1:** Clinical characteristics of 97 patients

Parameters	Cachexia (N = 47)	Non-cachexia (N = 50)	*p* Value
Ages (years)	69.80 ± 5.41	64.60 ± 8.18	0.114
Sex (M/F)	27/20	24/26	0.328
BMI (kg/m^2^)	18.38 ± 1.03	23.03 ± 0.90	**<0.001**
Weight Loss (kg)	5.50 ± 1.96	0.46 ± 0.74	**<0.001**
HB (g/dl)	112.40 ± 27.82	124.75 ± 22.58	0.158
WBC (10^9^/L)	6.23 ± 1.29	5.31 ± 0.89	0.078
Neu (10^9^/L)	3.72 ± 0.98	3.24 ± 0.68	0.132
Lymph (10^9^/L)	1.76 ± 0.60	1.79 ± 0.46	0.902
Alb (g/L)	40.67 ± 3.86	42.20 ± 2.86	0.165
PAb (mg/L)	186.21 ± 32.94	247.28 ± 72.63	**0.039**
TRF (g/L)	2.13 ± 0.41	2.50 ± 0.33	**0.045**
TC (mmol/L)	4.35 ± 0.93	4.91 ± 1.07	0.215
TG (mmol/L)	1.11 ± 0.21	1.33 ± 0.26	0.076
FFA (mmol/L)	0.53 ± 0.88	0.40 ± 0.14	**0.048**
HDL (mmol/L)	1.22 ± 0.34	1.01 ± 0.30	0.135
LDL (mmol/L)	2.70 ± 0.82	2.82 ± 0.77	0.743
Apo A(g/L)	1.23 ± 0.37	1.27 ± 0.31	0.872
Apo B(g/L)	0.89 ± 0.22	0.98 ± 0.25	0.323
Apo E(mg/L)	38.66 ± 6.10	58.83 ± 18.92	**0.006**
CRP (mg/dl)	2.43 ± 2.96	1.97 ± 1.89	0.683
TNFα (mmol/L)	10.21 ± 4.31	7.91 ± 3.93	0.228
IL6 (mmol/L)	6.67 ± 1.69	3.85 ± 1.41	**0.012**

Values in bold indicate statistically significant differences (*p* < 0.05).

Alb, Albumin; Apo A, Apolipoprotein A; Apo B, Apolipoprotein B; Apo E, Apolipoprotein E; BMI, Body mass index; CRP, C-reactive protein; HB, Hemoglobin; FFA, Free fatty acid; HDL, High-density lipoprotein; IL-6, Interleukin 6; LDL, Low-density lipoprotein; Lymph, Lymphocyte count; Neu, Neutrophil; PAb, Prealbumin; TC, Total cholesterol; TG, Tri-glyceride; TNF-α, Tumor Necrosis Factor-α; TRF, Transferrin; WBC, White blood cell.

### CircAMOTL1 promotes lipolysis and WAT browning in adipocytes

**Figure 2:**
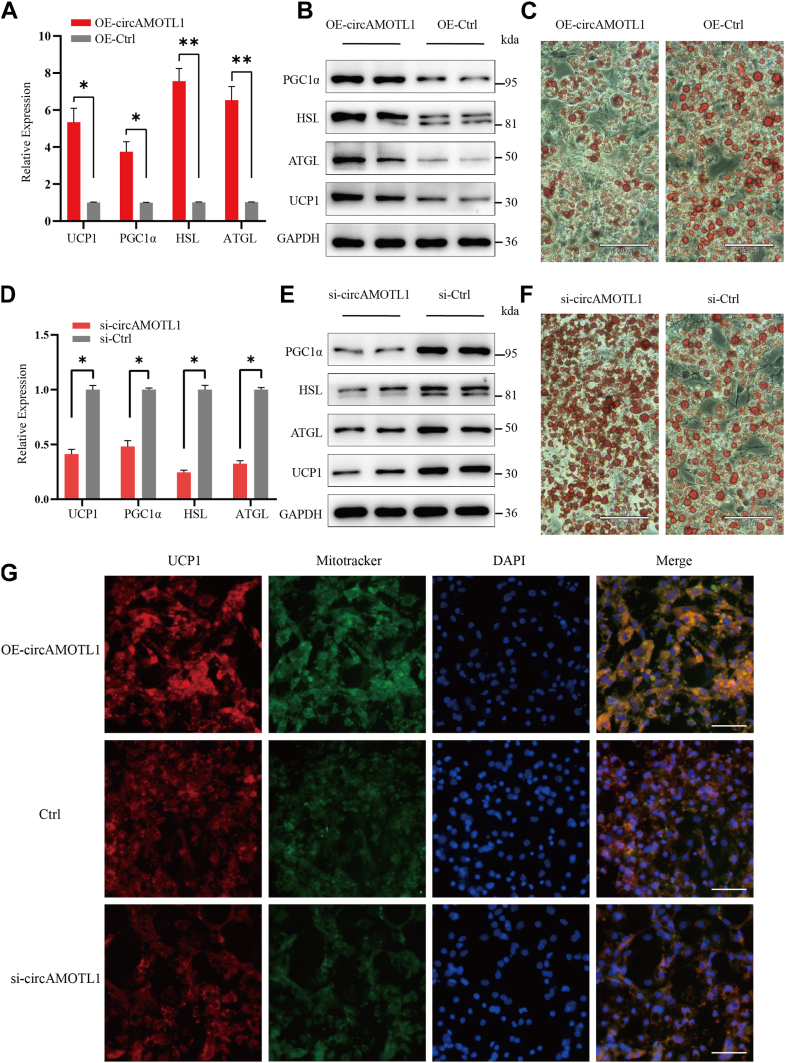
**CircAMOTL1 promotes lipolysis and WAT browning in adipocytes.***A* and *B*, qPCR and Western blot analysis of the expression of adipose-related markers in adipocytes without/with overexpression of circAMOTL1. *C*, ORO staining of lipid accumulation in adipocytes without/with overexpression of circAMOTL1 (Scale bar: 100 μm). *D* and *E*, qPCR and Western blot analysis of the expression of adipose-related markers in adipocytes without/with knockdown of circAMOTL1. *F*, ORO staining of lipid accumulation in adipocytes without/with knockdown of circAMOTL1 (Scale bar: 100 μm). *G*, IF of differentiated adipocytes staining for mitochondria (MitoTracker, *green*), anti-UCP1 (*red*), and nuclei (DAPI, *blue*) (Scale bar: 100 μm).

To elucidate the role of circAMOTL1 in adipocyte metabolism, preadipocytes were transfected with circAMOTL1 overexpression vectors, followed by induction of differentiation. Quantitative PCR analysis revealed a ∼3-fold increase in circAMOTL1 expression by day 6 of differentiation in the overexpression group compared to controls, while levels of linear AMOTL1 mRNA remained unchanged (Fig. S1*A*). The expression of key metabolic regulators-including HSL, ATGL, UCP1 and PGC-1α was assessed by qPCR and western blotting (Fig. 2, *A* and *B*). Overexpression of circAMOTL1 enhanced lipolysis and promoted the browning of adipocytes by day 6, as evidenced by ORO staining (Fig. 2*C*). Consistently, levels of FFAs (FFA) released into the culture medium were elevated following circAMOTL1 overexpression (Fig. S1*B*), indicative of increased lipolytic activity. CircAMOTL1 overexpression did not affect expression of adipogenic markers (PPARγ, C/EBPα), which indicated that circAMOTL1 regulates metabolic remodeling rather than adipocyte differentiation (Fig. S2). To further determine whether circAMOTL1 was necessary for adipocyte metabolic function, adipocytes were transfected with siRNAs specifically targeting the back-splice junction of circAMOTL1, thereby selectively silencing the circular transcript without affecting linear AMOTL1 mRNA expression. One siRNA achieved approximately 70% knockdown efficiency (Fig. S1*C*). Concordantly, expression of lipolysis markers (HSL, ATGL) and browning markers (UCP1, PGC-1α) were markedly reduced upon circAMOTL1 silencing (Fig. 2, *D* and *E*). ORO staining further demonstrated diminished lipolysis and browning in circAMOTL1-depleted adipocytes (Fig. 2*F*). In parallel, FFA concentrations in the culture medium were significantly decreased (Fig. S1*D*), reinforcing the role of circAMOTL1 in promoting lipolysis. Moreover, MitoTracker staining revealed abundant mitochondrial content in differentiated cells expressing UCP1, and mitochondrial abundance varied according to circAMOTL1 expression levels, providing additional evidence that circAMOTL1 facilitates the browning of WAT (Fig. 2*G*).

### CircAMOTL1 acts as a sponge for miR-211-5p

**Figure 3:**
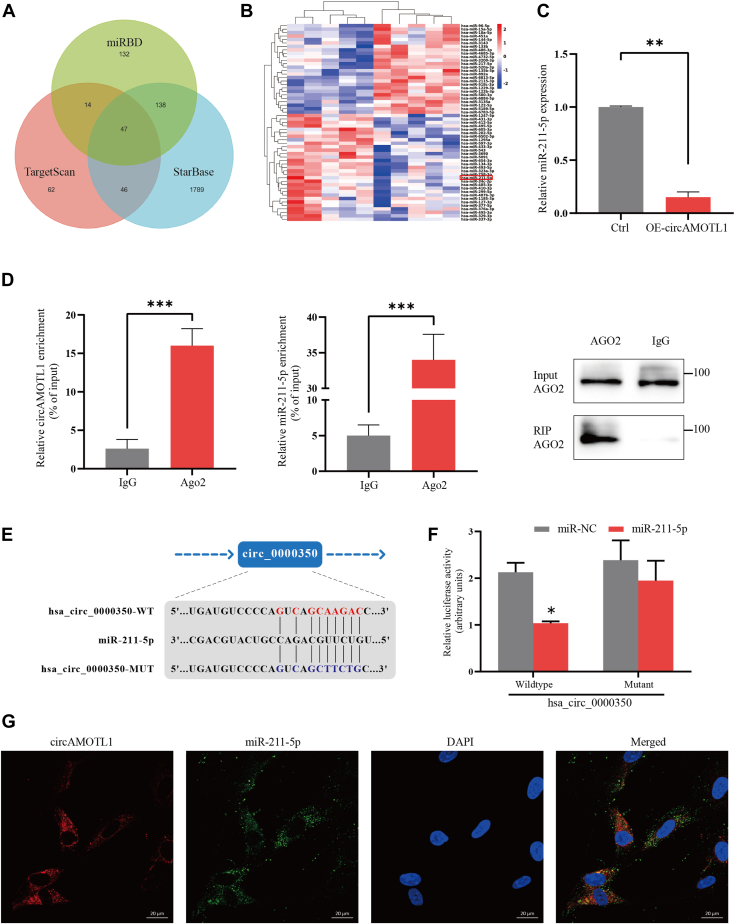
**CircAMOTL1 acts as a sponge for miR-211-5p.***A*, Venn diagram of circAMOTL1 targeted miRNAs, predicted by TargetScan, StarBase, and miRDB databases. *B*, schematic illustration exhibiting the heatmap of the differentially expressed miRNAs. *C*, the expression of miR-211-5p after overexpression of circAMOTL1 in adipocytes. *D*, RIP analysis of circAMOTL1 and miR-211-5p in adipocytes using antibodies against AGO2. Western blotting analysis of immunoprecipitated AGO2 protein is shown. *E*, schematic of the predicted miR-211-5p binding site on circAMOTL1. *F*, luciferase activity of wild-type or mutated circAMOTL1 in 293T cells after co-transfection with miR-211-5p or miRNA control. *G*, colocalization between miR-211-5p and circAMOTL1 was observed by RNA FISH in adipocytes. The nuclei were stained with DAPI (Scale bar: 20 μm).

To investigate the subcellular localization of circAMOTL1, we performed qPCR on nuclear and cytoplasmic RNA fractions. The results revealed that circAMOTL1 was predominantly enriched in the cytoplasm of adipocytes (Fig. S3). Given previous reports that cytoplasmic circRNAs often function as miRNA sponges to modulate gene expression (15, 16), we sought to identify candidate miRNAs potentially interacting with circAMOTL1. Differentially expressed miRNAs were analyzed using the miRDB, TargetScan, and StarBase prediction algorithms, yielding 47 miRNAs with putative binding sites within the circAMOTL1 sequence (Fig. 3*A*). We next integrated this bioinformatic prediction with our RNA sequencing data to examine miRNA expression profiles in cachectic *versus* non-cachectic adipose tissue. The analysis identified 128 upregulated and 167 downregulated miRNAs in the cachexia group (|log_2_FC| > 1, *p* < 0.05) (Fig. 3*B*). Among these, miR-211-5p emerged as a promising candidate, and its inverse correlation with circAMOTL1 was confirmed by qPCR (Fig. 3*C*). Considering the cytoplasmic localization of circAMOTL1, we hypothesized that it may participate in a ceRNA network *via* interaction with AGO2, a central component of the RNA-induced silencing complex (17). RIP using an anti-AGO2 antibody revealed significant enrichment of both circAMOTL1 and miR-211-5p in AGO2 complexes (*p* < 0.001; Fig. 3*D*), supporting direct interaction. To validate this interaction functionally, we conducted dual-luciferase reporter assays in 293T cells. Co-transfection of miR-211-5p mimics with wild-type circAMOTL1 luciferase reporter constructs resulted in about 40% reduction in luciferase activity, whereas mutant constructs lacking the miR-211-5p binding site showed no significant change (Fig. 3, *E* and *F*). Furthermore, FISH demonstrated cytoplasmic co-localization of circAMOTL1 and miR-211-5p in adipocytes (Fig. 3*G*). Collectively, these findings establish that circAMOTL1 directly binds to miR-211-5p, functioning as a molecular sponge.

### TET2 is a downstream target of miR-211-5p and circAMOTL1 and is involved in lipolysis and browning

**Figure 4:**
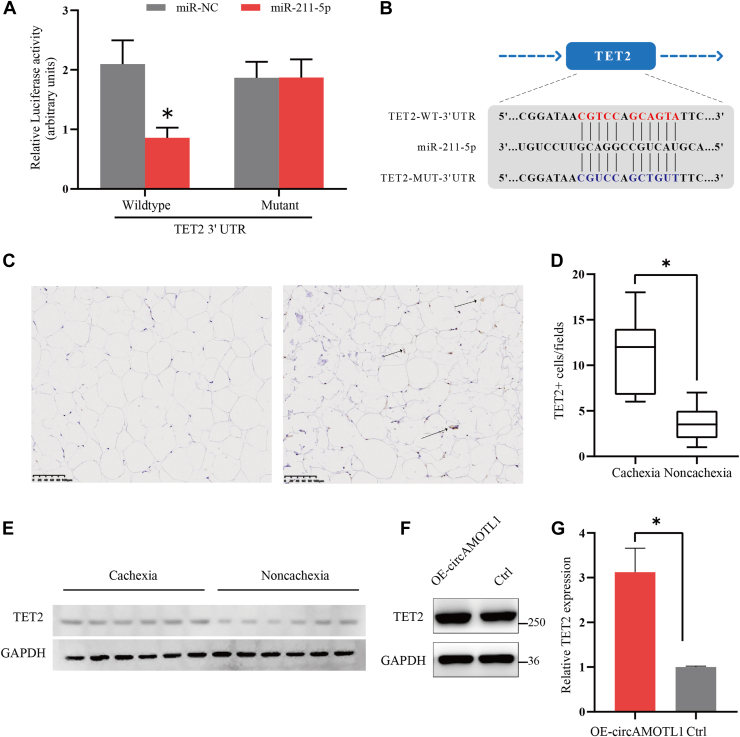
**TET2 is a downstream target of miR-134-5p and circAMOTL1.***A*, schematic of the predicted miR-211-5p binding site in the TET2 3′ UTR. *B*, Luciferase activity of wild-type or mutated TET2 3′ UTR in 293T cells after co-transfection with miR-211-5p or miRNA control. *C*, representative IHC staining of TET2 expression in adipose tissues from patients without/with cachectic (Scale bar: 100 μm). *Arrows* indicate cells expressing TET2. *D*, quantification of TET2-positive adipocytes in each field. *E*, WB analysis of cachectic and non-cachectic adipose tissue samples. *F* and *G*, WB and qPCR showed expression of TET2 after overexpression of circAMOTL1 in adipocytes.

To investigate the downstream effectors of the circAMOTL1/miR-211-5p axis, we utilized the TargetScan algorithm to predict potential target genes of miR-211-5p. Among the candidates, TET2 emerged as a putative target. To validate this interaction, we constructed luciferase reporter vectors containing either the wild-type or mutant TET2 3′UTR harboring the predicted miR-211-5p binding site (Fig. 4*A*). In 293 T cells, co-transfection with miR-211-5p mimics significantly reduced the luciferase activity of the wild-type TET2 3′UTR reporter, whereas no suppression was observed with the mutant construct (Fig. 4*B*), confirming the direct targeting of TET2 by miR-211-5p. To assess the relevance of TET2 in adipose tissue under cachectic conditions, we performed IHC staining in adipose specimens from patients with or without cachexia. IHC analysis revealed elevated TET2 expression in adipose tissues of cachectic individuals (Fig. 4, *C* and *D*), a finding further supported by WB analysis of independent adipose tissue samples (Fig. 4*E*). Furthermore, overexpression of circAMOTL1 in adipocytes significantly increased TET2 levels (Fig. 4, *F* and *G*), suggesting that circAMOTL1 may regulate TET2 expression *via* miR-211-5p.

**Figure 5:**
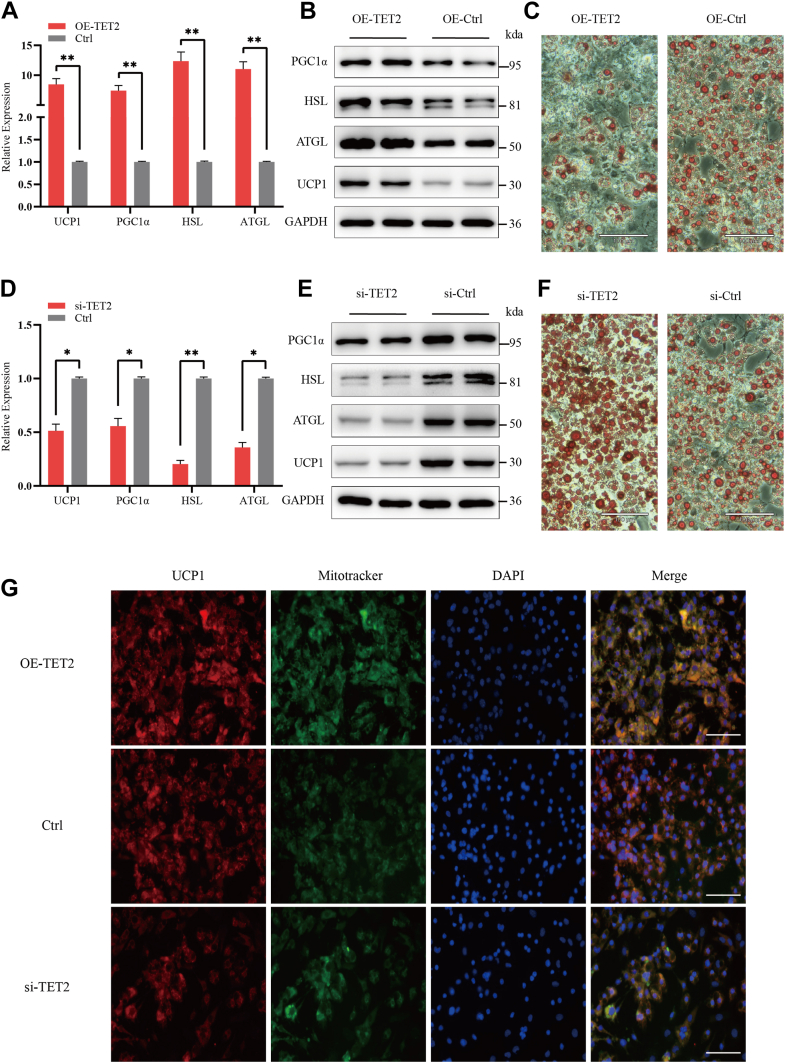
**TET2 promotes lipolysis and WAT browning in adipocytes.***A* and *B*, qPCR and Western blot analysis of the expression of adipose-related markers in adipocytes without/with overexpression of TET2. *C*, ORO staining of lipid accumulation in adipocytes without/with overexpression of TET2 (Scale bar: 100 μm). *D* and *E*, qPCR and Western blot analysis of the expression of adipose-related markers in adipocytes without/with knockdown of TET2. *F*, ORO staining of lipid accumulation in adipocytes without/with knockdown of TET2 (Scale bar: 100 μm). *G*, IF of differentiated adipocytes staining for mitochondria (MitoTracker, *green*), anti-UCP1 (*red*), and nuclei (DAPI, *blue*) (Scale bar: 100 μm).

To delineate the role of TET2 in adipocyte metabolism, we generated a TET2 overexpression construct and designed siRNAs targeting TET2, which were subsequently transfected into adipocytes. Following 8 days of induction and differentiation, cells were harvested for qPCR, WB and ORO staining. Transfection with the TET2 overexpression vector led to a marked upregulation of lipolysis- and browning-associated genes (Fig. 5*A*), a finding corroborated by western blotting (Fig. 5*B*). ORO staining further revealed a significant reduction in lipid droplet accumulation upon TET2 overexpression (Fig. 5*C*). Conversely, TET2 silencing *via* siRNA resulted in the suppression of lipolysis and browning markers at both the mRNA and protein levels (Fig. 5, *D* and *E*), accompanied by a pronounced increase in lipid droplet accumulation, as visualized by ORO staining (Fig. 5*F*). FFA levels in the culture medium were significantly decreased following TET2 knockdown, whereas TET2 overexpression led to increased FFA release (Fig. S4, *A* and *B*), further supporting its role in promoting lipolytic activity. In addition, MitoTracker staining revealed enhanced mitochondrial abundance in differentiated adipocytes expressing UCP1, with mitochondrial content varying in accordance with TET2 expression levels, which further proved TET2 promotes the browning of WAT (Fig. 5*G*). These results collectively indicate that TET2 plays a critical role in promoting lipolysis and driving the browning of WAT.

### CircAMOTL1 promotes lipolysis and browning by relieving the suppression effects of miR-211-5p on TET2

**Figure 6:**
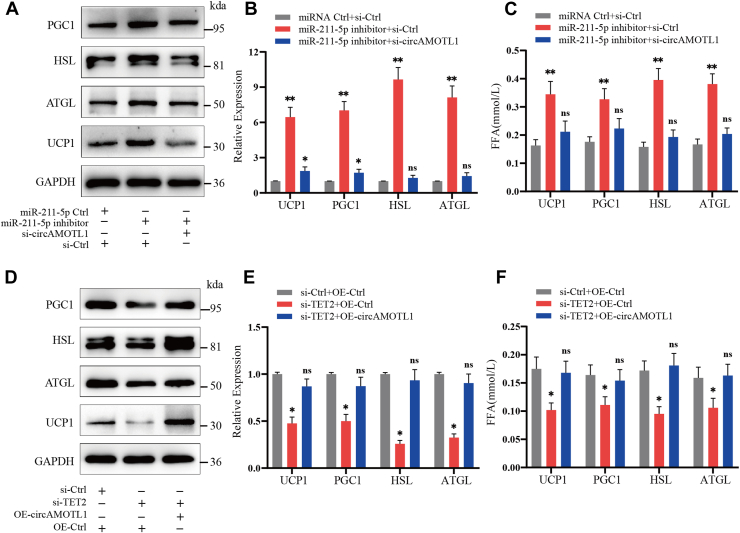
**CircAMOTL1 promotes lipolysis and WAT browning by relieving the suppression effects of miR-211-5p on TET2.***A–C*, qPCR, Western blot, and FFA concentration assay were performed in adipocytes treated with negative control, miR-211-5p inhibitor, circAMOTL1 siRNA + miR-211-5p inhibitor. *D–F*, qPCR, Western blot and FFA concentration assay were performed in adipocytes treated with negative control, TET2 siRNA, circAMOTL1 overexpression vector + TET2 siRNA.

To determine whether circAMOTL1 exerts its regulatory effects on adipocytes *via* miR-211-5p, we performed qPCR, Western blotting, and FFA concentration assay following transfection with a miR-211-5p inhibitor. Suppression of miR-211-5p expression significantly enhanced lipolysis and promoted browning in adipocytes. However, co-transfection with circAMOTL1 siRNA effectively abrogated these effects, reversing the upregulation of lipolytic and browning markers induced by miR-211-5p inhibition (Fig. 6, *A*–*C*). These findings suggest that circAMOTL1 modulates adipocyte metabolism by acting through miR-211-5p. We next examined whether TET2 is required for the metabolic regulation mediated by the circAMOTL1/miR-211-5p axis. Notably, overexpression of circAMOTL1 substantially rescued the impaired lipolysis and browning phenotype resulting from TET2 knockdown in adipocytes (Fig. 6, *D*–*F*), supporting a downstream role for TET2. Collectively, these results establish that circAMOTL1 functions as a molecular sponge for miR-211-5p, thereby modulating TET2 expression and promoting adipocyte lipolysis and browning through the ceRNA mechanism.

### CircAMOTL1 promotes lipolysis and WAT browning *in vivo*

**Figure 7:**
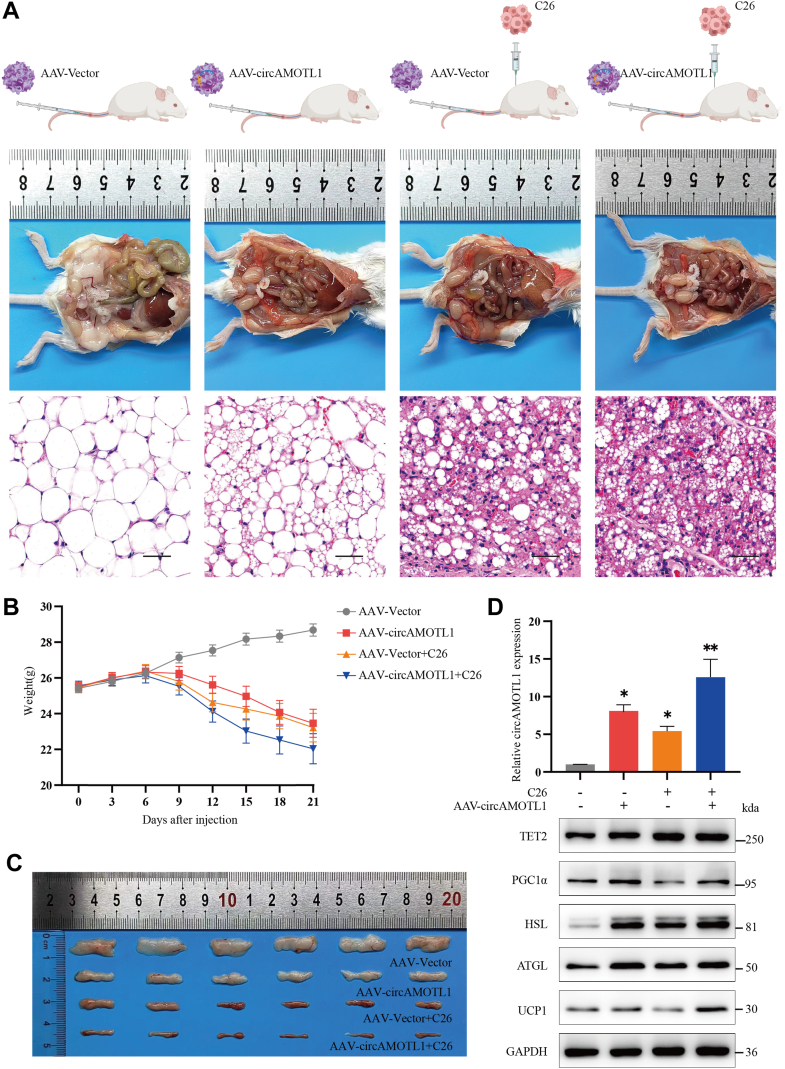
**CircAMOTL1 promotes lipolysis and WAT browning *in vivo*.***A*, schematic diagram, representative images, and H&E staining of subcutaneous adipose tissues in four different groups of mice. *B*, weight changes in four different groups of mice after injections. *C*, comparison of harvested subcutaneous WAT from the four experimental groups (from *top* to *bottom*, AAV-Vector, AAV-circAMOTL1, AAV-Vector + C26, and AAV-circAMOTL1 + C26). *D*, the expression of circAMOTL1 and protein levels of HSL, ATGL, UCP1, PGC1α and TET2 in adipose tissues from four different groups were analyzed.

To further assess the functional role of circAMOTL1 *in vivo*, a recombinant AAV carrying a circAMOTL1 overexpression construct (AAV-circAMOTL1) was generated and systemically administered *via* tail vein injection in BALB/c mice. A tissue-specific promoter was incorporated into the AAV vector to ensure selective expression of circAMOTL1 in adipose tissue. To model cancer-associated cachexia, C26 colon carcinoma cells were subcutaneously inoculated into the right flank of a separate cohort of mice. Both AAV-circAMOTL1-treated and cachectic mice exhibited markedly reduced subcutaneous adipose tissue compared to AAV control mice (Fig. 7*A*). Longitudinal monitoring revealed significant weight loss in mice treated with AAV-circAMOTL1, C26 cells, or both, beginning on day 6 post-injection (Fig. 7*B*). Tumor tissues collected from all mice showed no significant differences in tumor volume or weight between groups, indicating that circAMOTL1 overexpression did not alter tumor growth *in vivo*. Harvested subcutaneous adipose tissues from four experimental groups (AAV-Vector, AAV-circAMOTL1, AAV-Vector + C26, and C26 + AAV-circAMOTL1) were compared, and macroscopic evaluation confirmed reduced subcutaneous fat mass in C26- or/and AAV-circAMOTL1-treated groups (Fig. 7*C*). H&E staining demonstrated that AAV-circAMOTL1-treated mice displayed similar adipocyte morphology comparable to that observed in cachectic mice, characterized by shrunken, multilocular adipocytes (Fig. 7*A*). qPCR revealed an ∼8-fold increase in circAMOTL1 expression in AAV-circAMOTL1-injected mice and ∼5-fold elevation in cachectic mice relative to controls (Fig. 7*D*). Western blot analysis showed upregulation of TET2, HSL, ATGL, UCP1, and PGC1α proteins in AAV-circAMOTL1-treated mice, consistent with enhanced lipolytic and thermogenic activity. Consistent with our *in vitro* findings, AAV-mediated circAMOTL1 overexpression increased TET2 levels in adipose tissue by sequestering miR-211-5p and thereby relieving its repression of TET2. Interestingly, although TET2, HSL, and ATGL were also elevated in cachectic mice, UCP1 and PGC1α levels did not differ significantly from controls (Fig. 7*D*). Together, these findings indicate that circAMOTL1 overexpression robustly promotes lipolysis and browning of WAT *in vivo*.

## Discussion

This study identifies circAMOTL1 as a key regulatory molecule in adipose tissue remodeling during cancer cachexia and elucidates its mechanistic role in promoting lipolysis and browning through a miR-211-5p/TET2 axis. By integrating whole transcriptomic sequencing, patient-derived adipose samples, *in vitro* adipocyte models, and *in vivo* murine experiments, we demonstrate that circAMOTL1 is significantly upregulated in the subcutaneous WAT of cachectic patients. Its expression positively correlates with weight loss severity and detects cachexia with high diagnostic accuracy. Functional studies revealed that circAMOTL1 promotes adipocyte lipolysis and browning, partly by acting as a ceRNA for miR-211-5p, thereby relieving the repression of TET2, a downstream effector involved in metabolic remodeling. These findings provide mechanistic insight into how a specific circRNA contributes to the pathological fat loss in cachexia and suggest that circAMOTL1 may serve as a novel biomarker and potential therapeutic target. Our work adds to the growing evidence linking non-coding RNA regulation to metabolic dysfunction in cancer-associated cachexia and highlights a new molecular axis that could be targeted to mitigate adipose tissue atrophy in affected patients.

Recent advances in cachexia research have significantly improved our understanding of the complex interplay between metabolic dysfunction, systemic inflammation, and cancer progression. Traditionally, muscle wasting was considered the predominant feature of cachexia; however, growing evidence has proved that adipose tissue loss acts as an equally critical hallmark of this syndrome (18). Enhanced lipolysis, reduced adipogenesis, and a shift towards thermogenic remodeling, commonly termed adipose tissue browning, have been widely reported as significant contributors to adipose tissue depletion in cachexia (19). Notably, recent studies have elucidated the molecular pathways involved, implicating inflammatory cytokines such as IL-6, TNF-α as key regulators (20). These cytokines activate catabolic pathways that accelerate adipocyte lipolysis and inhibit adipocyte differentiation, ultimately leading to profound fat loss and metabolic derangements. Moreover, signaling pathways mediated by β-adrenergic receptors have emerged as essential mediators of adipocyte browning and energy expenditure in cachexia (21). Targeting these inflammatory and metabolic pathways thus presents promising therapeutic avenues to mitigate cachexia-associated adipose tissue wasting and improve patient outcomes.

Parallel to these metabolic insights, non-coding RNAs, including miRNAs, lncRNAs, and circRNAs, have increasingly been recognized for their regulatory roles in tissue remodeling and metabolic dysfunction. These non-coding RNAs modulate gene expression post-transcriptionally and translationally, exerting profound effects on cellular metabolism, inflammation, and differentiation. For instance, miRNAs such as miR-29a-3p and miR-16-5p have been implicated in regulating pathways critical to lipolysis and adipogenesis, while lncRNA-NEAT1 and lncRNA-HNSCR have been shown to influence adipocyte differentiation and inflammation through interactions with RNA-binding proteins or other non-coding RNAs (22, 23, 24). CircRNAs, characterized by their stability and tissue-specific expression, represent a particularly promising class of molecules for biomarker discovery and therapeutic targeting. Recent studies have begun to elucidate their roles in adipose tissue biology, with example such as circPPARG, regulated intramuscular fat deposition by promoting adipogenesis and suppressing lipolysis, revealing a novel non-canonical regulatory mechanism of PPARG beyond its transcriptional role (25). Our identification of circAMOTL1 as a regulatory node in the miR-211-5p/TET2 axis significantly expands this emerging narrative, providing novel insights into circRNA-mediated regulation of adipose tissue dynamics in cachexia.

Recent studies have demonstrated that circAMOTL1 plays a significant role in different types of cancer and cardiovascular diseases through diverse molecular mechanisms. For instance, circAMOTL1 has been implicated in diabetic myocardial fibrosis by promoting cardiac fibroblast proliferation and collagen deposition, primarily through interactions with the RNA-binding EIF4A3, thereby stabilizing the expression of MARCKS (26). In cancer biology, circAMOTL1 facilitates oncogenesis by acting as a molecular sponge for miRNAs or by regulating protein interactions. Notably, in breast cancer, circAMOTL1 promotes tumor progression by enabling the nuclear translocation of oncogenic proteins like c-MYC and AKT, subsequently activating downstream genes associated with tumor growth and malignancy (27). In prostate cancer, circAMOTL1 serves a tumor-suppressive role by sponging miR-193a-5p, thereby modulating the expression of target genes involved in cell proliferation and migration (27). Furthermore, research has indicated a critical role of circAMOTL1 in wound healing processes, where it influences cell proliferation and migration by regulating signaling pathways involving STAT3 (28). Collectively, these findings revealed the multifaceted functions of circAMOTL1, emphasizing its significance in cellular processes such as proliferation, fibrosis, and inflammation, and reinforcing its potential as a biomarker and therapeutic target across a spectrum of human diseases.

Recent studies have positioned miR-211-5p as a critical regulatory microRNA in cancer biology and metabolism. In melanoma, its expression is closely associated with tumor progression and clinical outcomes. Notably, miR-211-5p is highly expressed in normal melanocytes but becomes downregulated in dedifferentiated melanoma cells. Paradoxically, elevated miR-211-5p expression within metastatic lesions has been correlated with poor patient survival, potentially owing to its role in modulating glucose metabolism, proptosis, and the tumor immune microenvironment *via* direct targeting of GNA15. Furthermore, miR-211-5p can be horizontally transferred *via* exosomes from invasive tumor subclones to adjacent melanoma cells, thereby enhancing metastatic potential (29). In contrast, miR-211-5p exhibits tumor-suppressive properties in pancreatic cancer, where its reduced expression is associated with disease progression and adverse prognosis. Mechanistically, miR-211-5p impairs pancreatic tumor growth and metastasis by targeting BMP2, thereby limiting proliferative and invasive capacity (30). These findings underscore the dualistic role of miR-211-5p in cancer biology—acting either as a suppressor or facilitator of tumor progression depending on the cellular and molecular context—and highlight the need for further mechanistic investigation to delineate its context-specific functions.

Recent studies have significantly advanced our understanding of TET2's involvement in various biological and pathological processes. In clear cell renal cell carcinoma (ccRCC), TET2 expression negatively correlates with tumor progression and metastasis, uniquely positioning TET2 as a tumor suppressor gene in this context. Mechanistically, TET2 inhibits tumor progression by restraining glycolysis and the pentose phosphate pathway, processes that are typically dysregulated following von Hippel-Lindau (VHL) gene deficiency in ccRCC (31). Research highlights the requirement of TET2-mediated DNA demethylation for the epigenetic activation of genes critical for adipocyte differentiation. The iron-dependent catalytic activity of TET2, influenced by lysosome-mediated ferritinophagy, is particularly crucial in the early stages of adipogenesis. Disruptions in TET2 function or subcellular iron availability impair the demethylation of key adipogenic genes (32). It is revealed that TET2 deficiency in cancer cells disrupts lipid metabolism by downregulating HMGCS1 and the mevalonate pathway, making these cells vulnerable to statins. Statins induce apoptosis in TET2-deficient cells by depleting geranylgeranyl diphosphate (GGPP), which disrupts small GTPase function (33). Collectively, these findings emphasize TET2’s pivotal role across various cell types and diseases, providing opportunities for targeted therapeutic interventions.

In conclusion, our study uncovers a novel regulatory axis in which circAMOTL1 promotes lipolysis and browning of WAT by acting as a sponge for miR-211-5p, thereby relieving its repression of TET2. This circAMOTL1/miR-211-5p/TET2 axis was shown to operate in human adipose tissue, *in vitro* adipocyte models, and in a murine model of cancer cachexia, underscoring its biological relevance and potential translational significance. These findings highlight circRNAs as active regulators in metabolic disease and open new avenues for diagnostic and therapeutic strategies targeting cachexia-induced adipose tissue loss. However, several limitations warrant consideration. First, although we demonstrated mechanistic links in adipocyte cultures and *in vivo* models, whether circAMOTL1 plays a similar role in other metabolic tissues, such as muscle or liver, remains unclear. Second, the upstream regulatory mechanisms driving circAMOTL1 upregulation in cachexia were not addressed in this study. Finally, clinical translation would require validation in larger patient cohorts and assessment of the safety and feasibility of targeting this axis therapeutically. Future studies should aim to elucidate the broader RNA regulatory networks involving circAMOTL1 and investigate the feasibility of RNA-based interventions to attenuate adipose waste in cachexia.

In summary, through transcriptome-wide RNA sequencing, we identified a previously uncharacterized circular RNA, hsa_circ_0000350, derived from the AMOTL1 gene, that is significantly upregulated in subcutaneous adipose tissue of cachectic patients. Elevated circAMOTL1 expression was positively correlated with the extent of cachexia-associated weight loss. Mechanistically, circAMOTL1 functions as a ceRNA by sequestering miR-211-5p, thereby alleviating its repressive effect on TET2. This upregulation of TET2 contributes to enhanced lipolysis and browning. Collectively, our findings delineate a circAMOTL1/miR-211-5p/TET2 regulatory axis that governs lipid catabolism in adipocytes during cancer cachexia. These insights not only advance our understanding of circRNA-mediated metabolic regulation but also nominate circAMOTL1 as a potential biomarker and therapeutic target in the diagnosis and management of cancer cachexia.

## Experimental procedures

### Human adipose tissue specimens

Adipose tissue samples were obtained from patients diagnosed with gastrointestinal cancers undergoing radical surgical resection at the Department of General Surgery, Zhongshan Hospital. Patients were classified into cachexia and non-cachexia groups based on standardized diagnostic criteria, specifically >5% involuntary weight loss within 6 months preceding surgery. Adipose tissues were collected intraoperatively from the abdominal incision site, immediately frozen in liquid nitrogen, and subsequently stored at −80 °C. Ethical approval for this study was granted by the Ethics Committee of Zhongshan Hospital, Fudan University (Approval No. B2019-193R), and informed consents were collected from all participants. All human studies were performed in compliance with the principles of the Declaration of Helsinki.

### Cell culture and adipocyte differentiation

Immortalized murine preadipocyte cell lines were maintained in Dulbecco's modified Eagle medium (DMEM), supplemented with 10% fetal bovine serum (FBS), 100 U/ml penicillin, and 100 μg/ml streptomycin. Adipogenic differentiation was initiated upon reaching confluence by supplementing cells with differentiation medium containing insulin, dexamethasone, rosiglitazone, and 3-isobutyl-1-methylxanthine (IBMX). After 48 h, this medium was replaced with maintenance medium containing insulin (5 μg/ml) alone until mature adipocytes were obtained for subsequent experiments.

### RNA sequencing and bioinformatics analysis

Total RNA was extracted from adipose tissue samples utilizing TRIzol reagent following the manufacturer's protocol. Ribosomal RNA was depleted, and the sequencing libraries were constructed using the Illumina TruSeq library preparation kit. Sequencing was performed using the Illumina HiSeq platform. The raw sequencing dataset supporting the results of this study was deposited in the NCBI GEO database. The data is accessible through GEO: GSE174128. Differentially expressed circRNAs, miRNAs, and target mRNAs were identified based on |log2FC|>1 and adjusted *p*-values < 0.05. Bioinformatic databases including Circinteractome, miRDB, StarBase, and TargetScan were utilized to predict potential interactions between circRNAs, miRNAs, and target genes.

### Ribonuclease R treatment

To confirm the circular nature of circAMOTL1, total RNA was treated with RNase R at 37 °C for 30 min, followed by quantitative reverse transcription PCR (qRT-PCR) analysis comparing circRNA and linear RNA levels using divergent and convergent primers, respectively.

### Plasmids, siRNAs, and cell transfection

CircAMOTL1 expression vectors, miR-211-5p mimics and inhibitors, TET2 overexpression plasmids, and corresponding negative controls were synthesized by Gene Pharma. Three siRNAs targeting circAMOTL1 and TET2 were designed specifically to ensure selective knockdown. Transfections were conducted utilizing Lipofectamine 3000 or RNAiMAX (Invitrogen) according to manufacturer instructions, ensuring optimal transfection efficiency. All assays were conducted a minimum of three times.

### Quantitative reverse transcription PCR (qRT-PCR)

Total RNA extraction and reverse transcription were performed with TRIzol reagent and cDNA synthesis kits (Tiangen, Beijing, China), respectively. qRT-PCR was conducted using SYBR Green Master Mix (Tiangen) with primers specific to circAMOTL1, miR-211-5p, TET2, and internal controls GAPDH or U6. Primers used for real-time reactions were designed and synthesized by Sangon Biotech and are listed in the Additional file: Table S1. Relative expression was calculated using the 2^−ΔΔCt^ method, with each assay performed in triplicate.

### Western blotting analysis

Protein lysates extracted from cultured adipocytes or tissue samples were quantified using the bicinchoninic acid assay. Equal protein amounts were electrophoresed on SDS-PAGE gels and transferred to polyvinylidene fluoride membranes. After blocking, membranes were incubated overnight at 4 °C with primary antibodies against TET2 (Abcam, ab124297), adipose triglyceride lipase (ATGL) (Abcam, ab207799), hormone-sensitive lipase (HSL) (Cell signaling technology, 4107), uncoupling protein 1 (UCP1) (Cell signaling technology, 72298), peroxisome proliferator-activated receptor-gamma coactivator (PGC)-1alpha (Abcam, ab191838), and GAPDH. Protein bands were visualized with enhanced chemiluminescence reagents.

### Oil Red O staining

Mature adipocytes were fixed with 4% formaldehyde for 30 min, washed twice with PBS, and then stained with 0.3% Oil Red O (ORO) solution and washed three times with distilled water. Images were captured under a light microscope.

### Free fatty acid assay

Free fatty acid (FFA) concentration in conditioned medium was measured using a Non-esterified Free Fatty Acid Colorimetric Assay Kit (Thermo). The procedure was conducted according to the manufacturer’s instructions.

### Luciferase reporter assay

Luciferase reporter vectors containing wild-type or mutant circAMOTL1 sequences or the 3′-untranslated region (3′UTR) of TET2 with miR-211-5p binding sites were generated and verified by sequencing. Reporter plasmids were co-transfected with miR-211-5p mimics or control miRNA into HEK293T cells. After 48 h incubation, the cells were lysed, and the relative luciferase activity was exanimated using a Dual Luciferase Assay Kit (Promega) in accordance with the manufacturer’s protocol.

### Fluorescence *in situ* hybridization (FISH)

To verify subcellular localization, FISH assays were performed on adipocytes using Cy3-labeled circAMOTL1 probes and FITC-labeled miR-211-5p probes. Cells were fixed, and hybridized overnight at 37 °C. Nuclei were stained with 40,6-diamidino-2-phenylindole (DAPI). Slides were photographed with a confocal microscope (Leica).

### RNA immunoprecipitation (RIP)

RIP assays were performed to elucidate the molecular interactions between circAMOTL1 and miR-211-5p. Mature adipocytes were harvested and lysed in RIP lysis buffer containing protease inhibitors and RNase inhibitors. The lysates were incubated overnight at 4 °C with magnetic beads conjugated to an anti-Argonaute2 (AGO2, Abcam, ab57113) antibody or normal mouse IgG as a negative control. After stringent washing steps, RNA-protein complexes bound to magnetic beads were eluted, and RNA was extracted for subsequent qRT-PCR analysis. Enrichment of circAMOTL1 and miR-211-5p was determined relative to input controls, verifying specific interactions between circAMOTL1 and miR-211-5p.

### Immunohistochemistry (IHC)

IHC staining was carried out to assess the *in situ* expression levels and distribution patterns of TET2 in human adipose tissues. Paraffin-embedded tissue sections were deparaffinized, rehydrated, and antigen retrieval was performed using citrate buffer (pH 6.0) under high temperature. Following endogenous peroxidase inhibition with 3% hydrogen peroxide, sections were incubated with a blocking reagent. Slides were subsequently incubated overnight at 4 °C with primary antibodies specific to TET2, followed by incubation with horseradish peroxidase-conjugated secondary antibodies. Signal visualization was achieved using 3,3′-diaminobenzidine (DAB) substrate, and slides were counterstained with hematoxylin, dehydrated, and mounted for microscopic examination.

### Immunofluorescence (IF)

IF assays were conducted to determine the spatial localization and expression intensity of UCP1 in adipose tissues. Fresh-frozen adipose tissue sections were fixed with 4% paraformaldehyde, permeabilized with 0.5% Triton X-100, and blocked using 5% bovine serum albumin. Subsequently, sections were incubated overnight at 4 °C with primary antibodies against UCP1. After extensive washing, sections were incubated with fluorescently labeled secondary antibodies in a dark environment at room temperature. Nuclei were counterstained with DAPI. Immunofluorescent images were captured using a confocal microscope.

### Animal model and *in vivo* experiment

Six-week-old male BALB/c mice were purchased from Shanghai SLAC Laboratory Animal Corp and maintained under SPF conditions in a controlled environment of 20 to 22 °C, with a 12/12 h light/dark cycle. For *in vivo* circRNA overexpression, we used a recombinant adeno-associated viral (AAV) vector driven by the adipose-specific FABP4 promoter so that transgene expression was restricted to adipose tissue. Recombinant AAV containing circAMOTL1 constructs were administered intravenously to male BALB/c mice. Cachexia was induced by subcutaneous injection of colon-26 adenocarcinoma cells (C26). Construction of both mouse models was performed as previously described (34). Adipose tissues were collected after euthanasia for histological and molecular analyses, including qPCR and western blotting. All animal studies were performed in accordance with the guidelines provided by the Shanghai Medical Experimental Animal Care Commission.

### Statistical analysis

All statistical analyses were performed with SPSS 25.0 software. All experimental data were presented as mean ± standard deviation (SD). Statistical comparisons were made using Student’s *t* test or ANOVA, as appropriate. Correlation analyses were performed using Spearman’s method. Statistical significance is indicated as follows: *p* < 0.05 (∗), *p* < 0.01 (∗∗), and *p* < 0.001 (∗∗∗).

## Ethics approval and consent to participate

Ethical approval for this study was granted by the Ethics Committee of Zhongshan Hospital, Fudan University (Approval No. B2019-193R), and informed consents were collected from all participants.

## Data availability

Microarray data are deposited at the GEO database with the accession number GSE174128 (https://www.ncbi.nlm.nih.gov/geo/query/acc.cgi?acc=GSE174128). All other data is contained in the main text and Supporting Information.

## Supporting information

This article contains supporting information.

## Conflict of interest

The authors declare that they do not have any conflicts of interest with the content of this article.
