# Supplementary material for: CircAMOTL1 promotes adipose lipolysis and browning in cancer cachexia through miR-211-5p-mediated TET2 activation
Source: J Biol Chem. 2025 Dec 12;302(3):111052. doi: 10.1016/j.jbc.2025.111052 (PMC12969430; doi:10.1016/j.jbc.2025.111052)
Supplement: Supporting information [file mmc1.docx]

**
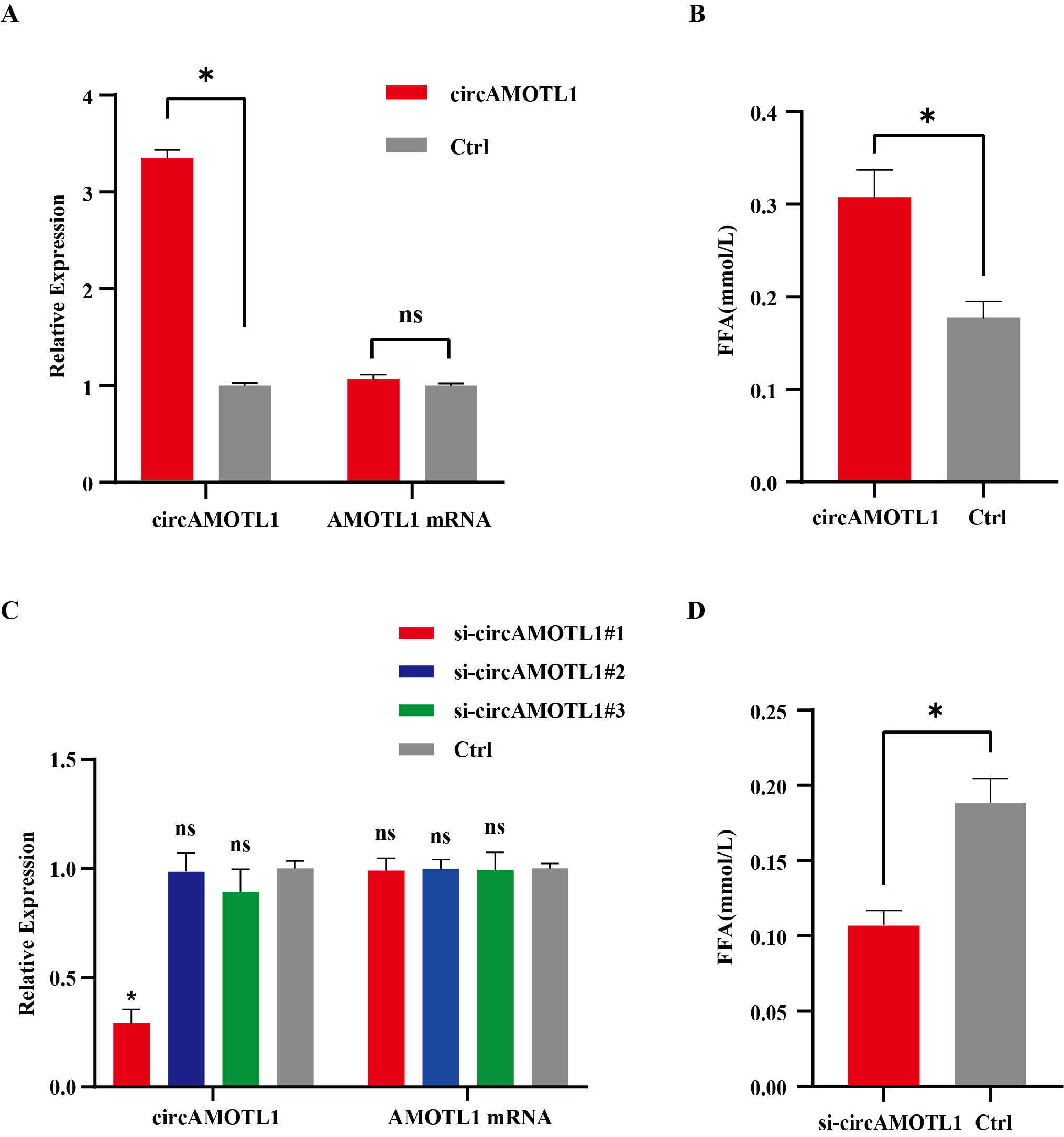
**

**FigS1.** Overexpression and knockdown efficiency of circAMOTL1 in adipocytes. (A) Relative circAMOTL1 and AMOTL1 mRNA expression with or without circAMOTL1 overexpression. (B) Concentration of FFA released in culture medium by adipocytes without/with overexpression of circAMOTL1. (C) Relative circAMOTL1 and AMOTL1 mRNA expression with or without circAMOTL1 downregulation. (D) Concentration of FFA released in culture medium by adipocytes without/with knockdown of circAMOTL1.

**
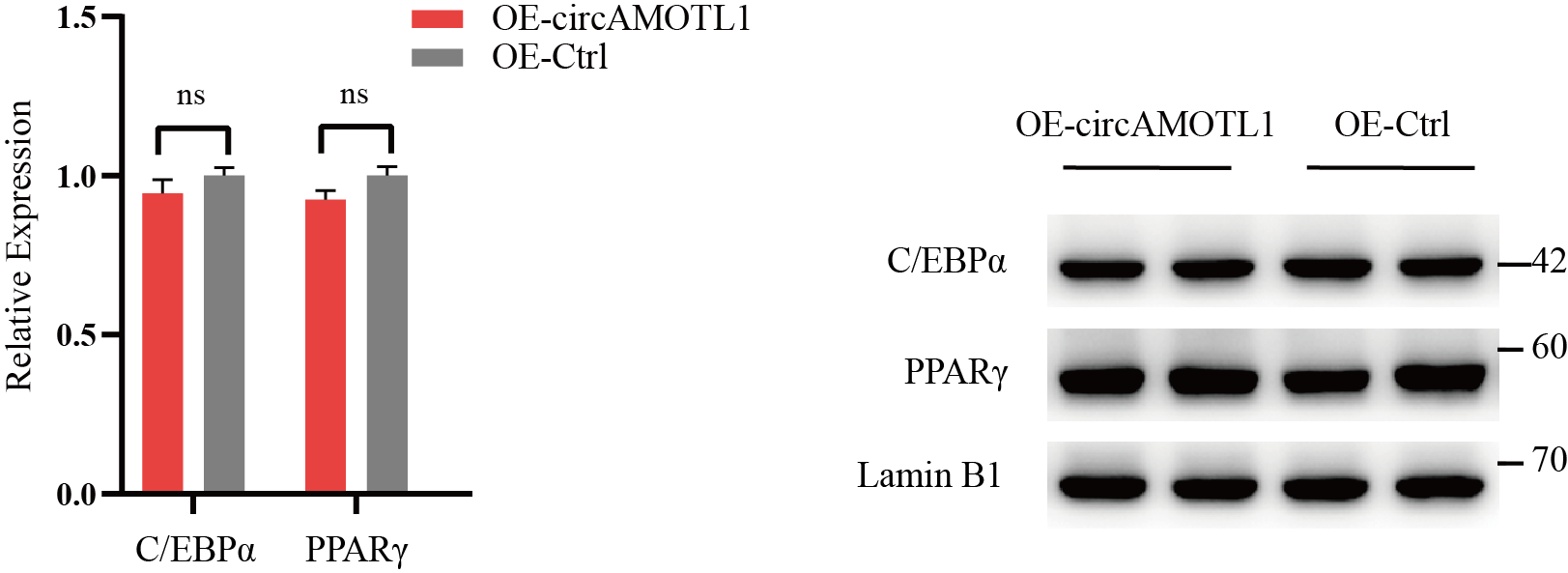
**

**FigS2.** qPCR and WB of adipogenic markers (PPARγ, C/EBPα) measured on day 6 of differentiation showed no significant changes, indicating circAMOTL1 does not alter adipogenic differentiation capacity.

**
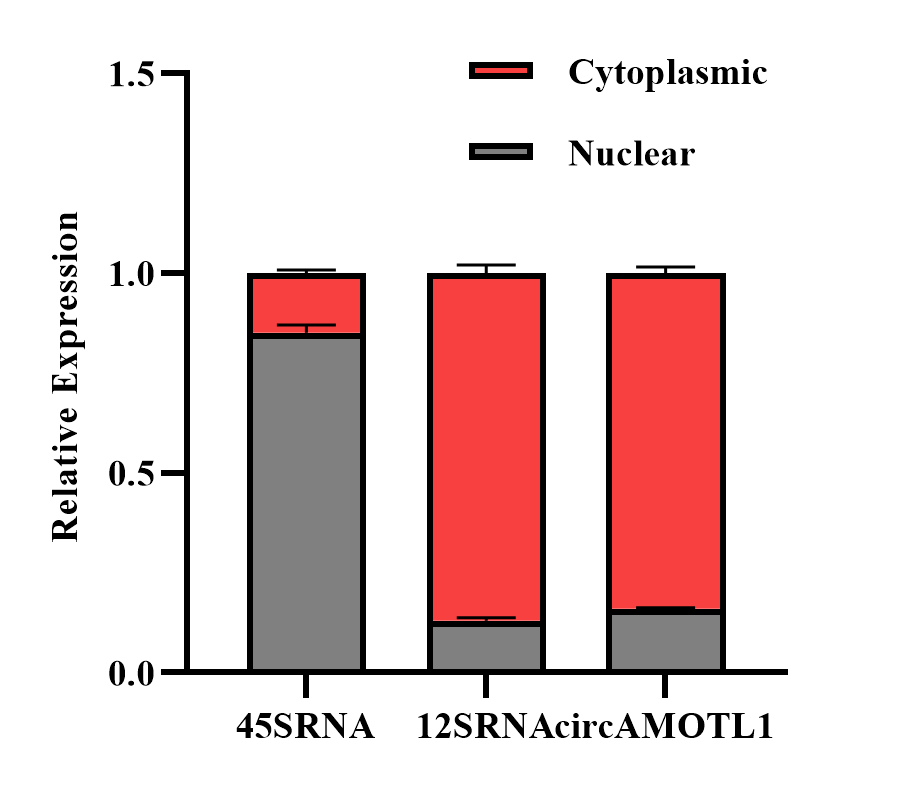
**

**FigS3.** qPCR analyses nuclear and cytoplasmic fractions from adipocytes. 12S rRNA served as positive controls in cytoplasm; 45S rRNA served as positive controls in nuclei.

**
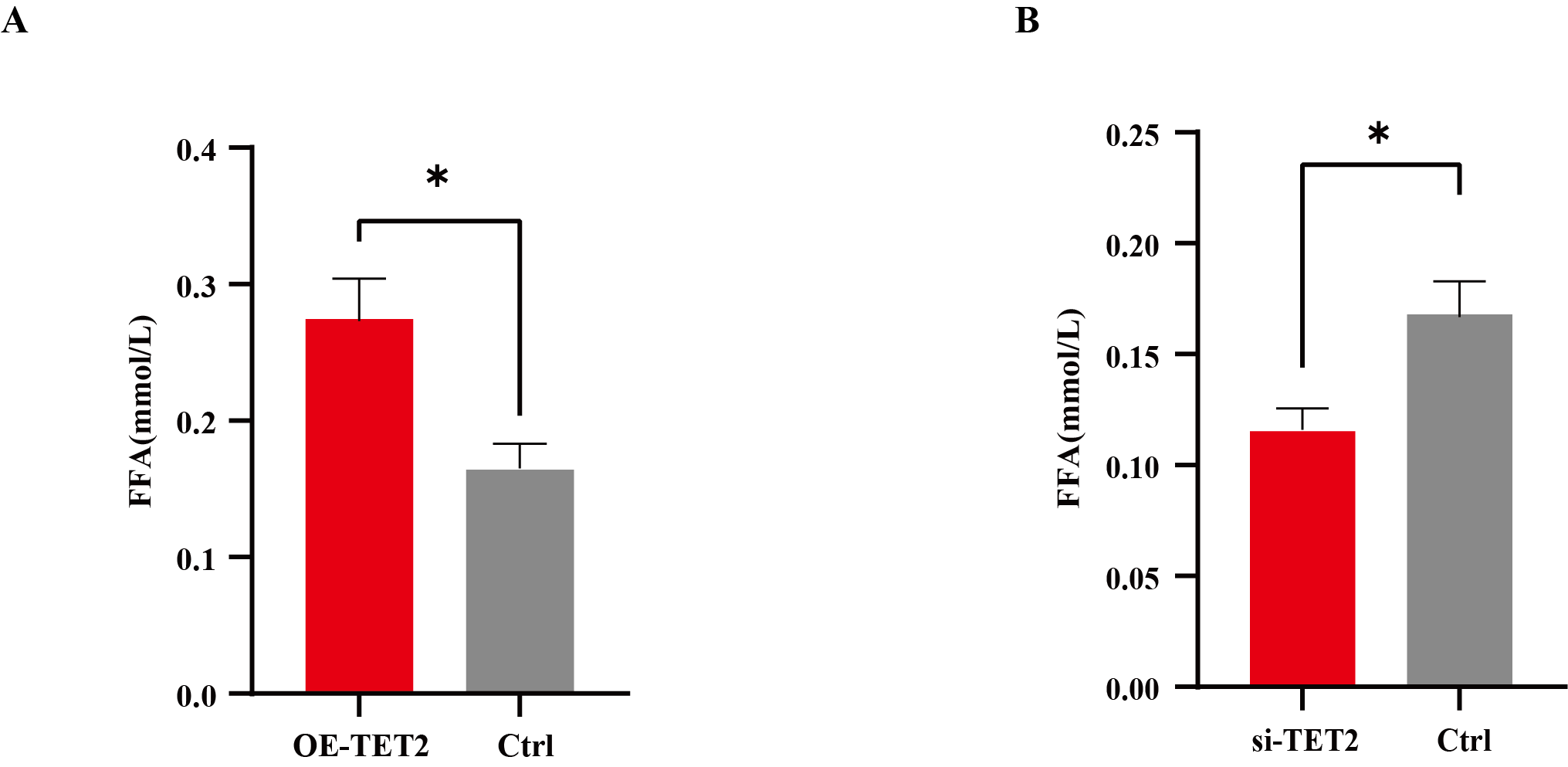
**

**FigS4.** (A) Concentration of FFA released in culture medium by adipocytes without/with overexpression of TET2. (B) Concentration of FFA released in culture medium by adipocytes without/with knockdown of TET2.

**
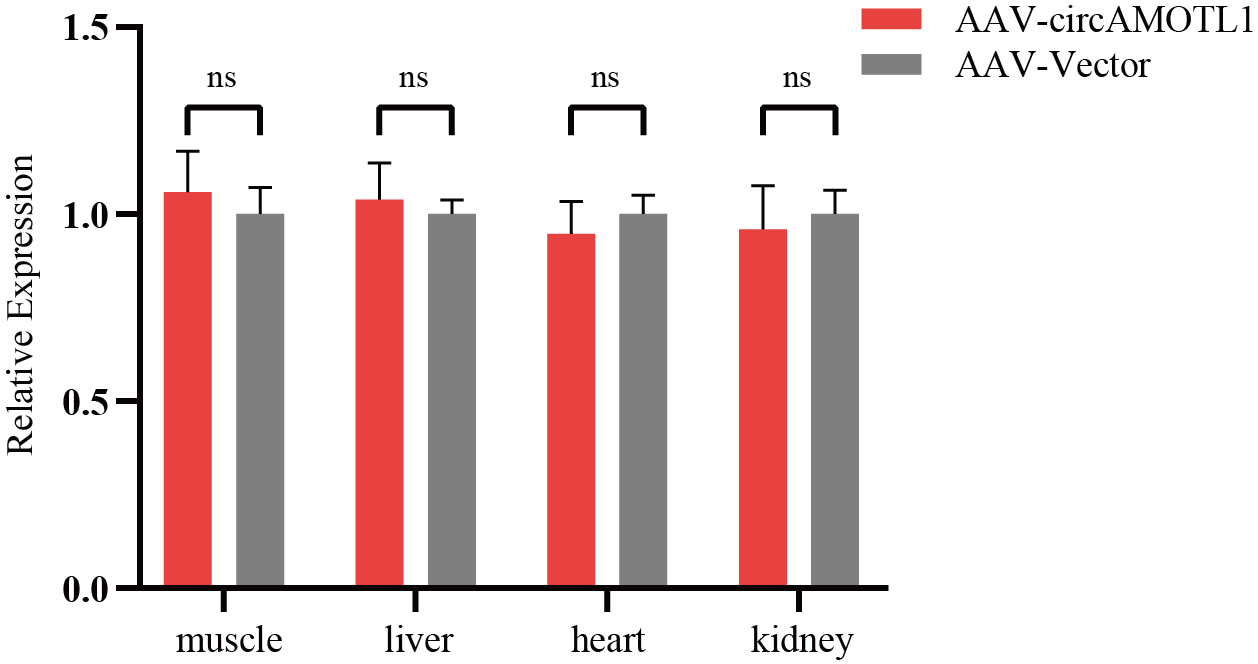
**

**FigS5.** No significant circAMOTL1 overexpression was detected in any non-adipose tissue, confirming adipose-restricted expression.

**Table S1. Primer Sequences used for qRT-PCR.**

| Name | Primer Sequences |
| --- | --- |
| TET2 | Forward:5′-CAGGACCCAGAAGTCCCTTC-3′ |
|  | Reverse:5′-GCTGGAGATGGTGGAGTTGA-3′ |
| HSL | Forward:5′-TGCTGGAGAAGGACAAGGAG-3′ |
|  | Reverse:5′-TCCTTGGCTGTTGTCTTGCT-3′ |
| ATGL | Forward:5′-GCTGGAGTTCCTGGAGAATG-3′ |
|  | Reverse:5′-AGGTCCTGGTGAGGAAGAGG-3′ |
| PGC1α | Forward:5′-TGAAGAGGCCCAGAAAGCTA-3′ |
|  | Reverse:5′-GTCGTTCACGTCAGCTTCCT-3′ |
| UCP1 | Forward:5′-CTGGCATCAGCCTTACCATC-3′ |
|  | Reverse:5′-GCCAGGATGAGGTCAAAGAA-3′ |
| AMOTL1 | Forward:5′-AGCTGGAGGAGGAGAAGGAC-3′ |
|  | Reverse:5′-TCCTCCTTCTCCTCCTTCTC-3′ |
| GAPDH | Forward: 5′-TTGCCCTCAACGACCACTTT-3′ |
|  | Reverse: 5′-TGGTCCAGGGGTCTTACTCC-3′ |
| U6 | Forward: 5′-ATTGGAACGATACAGAGAAGATT-3′ |
|  | Reverse: 5′-GGAACGCTTCACGAATTTG-3′ |
| miR-211-5p | 5′-GTCGTATCCAGTGCAGGGTCCGAGGTATT -3′ |
| si-circAMOTL1#1 | Forward: 5′-GGAUGAAGUUCAAGGACAATT-3′ |
|  | Reverse: 5′-UUGUCCUUGAACUUCAUCCTT-3′ |
| si-circAMOTL1#2 | Forward: 5′-CUGCUAUGAAGACCAGUAATT-3′ |
|  | Reverse: 5′-UUACUGGUCUUCAUAGCAGTT-3′ |
| si-circAMOTL1#3 | Forward: 5′-CCAGAAGUCCUGUUCUCAATT-3′ |
|  | Reverse: 5′-UUGAGAACAGGACUUCUGGTT-3′ |
| si-TET2#1 | Forward: 5′-GGACUGAUAUGAAGACUAATT-3′ |
|  | Reverse: 5′-UUAGUCUUCAUAUCAGUCCTT-3′ |
| si-TET2#2 | Forward: 5′-CUGUCAGUAUCAGGAUCAATT-3′ |
|  | Reverse: 5′-UUGAUCCUGAUACUGACAGTT-3′ |
| si-TET2#3 | Forward: 5′-CCAGAAGUCCUGUUCUCAATT-3′ |
|  | Reverse: 5′-UUGAGAACAGGACUUCUGGTT-3′ |
